# Effects of Temperature and pH on Recombinant Thaumatin II Production by *Pichia pastoris*

**DOI:** 10.3390/foods11101438

**Published:** 2022-05-16

**Authors:** Jewel Ann Joseph, Simen Akkermans, Jan F. M. Van Impe

**Affiliations:** BioTeC+, Chemical and Biochemical Process Technology and Control, Department of Chemical Engineering, KU Leuven, 9000 Ghent, Belgium; jewelann.joseph@kuleuven.be (J.A.J.); simen.akkermans@kuleuven.be (S.A.)

**Keywords:** *Pichia pastoris*, recombinant sweet protein, thaumatin, cultivation conditions

## Abstract

The sweet protein thaumatin is emerging as a promising sugar replacer in the market today, especially in the food and beverage sector. Rising demand for its production necessitates the large-scale extraction of this protein from its natural plant source, which can be limited in terms of raw material availability and production costs. Using a recombinant production technique via a yeast platform, specifically, *Pichia pastoris*, is more promising to achieve the product economically while maintaining batch-to-batch consistency. However, the bioproduction of recombinant proteins requires the identification of optimal process variables, constituting the maximal yield of the product of interest. These variables have a direct effect on the growth of the host organism and the secretion levels of the recombinant protein. In this study, two important environmental factors, pH, and temperature were assessed by cultivating *P. pastoris* in shake flasks to understand their influence on growth and the production levels of thaumatin II protein. The results from the pH study indicate that *P. pastoris* attained a higher viable cell density and secretion of protein at pH 6.0 compared to 5.0 when grown at 30 °C. Furthermore, within the three levels of temperatures investigated when grown at pH 6.0, the protein levels were the highest at 30 °C compared to 20 and 25 °C, whereas 25 °C exhibited the highest viable cell density. Interestingly, the trend observed from the qualitative effects of temperature and pH occurred in all the media that was investigated. These results broaden our understanding of how pH and temperature adjustment during *P. pastoris* cultivation aid in enhancing the production yields of thaumatin II prior to optimising the fed batch bioreactor operation.

## 1. Introduction

Recombinant DNA technology can be used to integrate foreign genes into a suitable host and express them to attain valuable products for different sectors. There is expected to be a growing demand to produce large quantities of industrial enzymes and pharmaceutical products, causing recombinant technology to create a multibillion-dollar market in a brief time. Recombinant proteins are used in a variety of fields, the most common of which are medical, academic, and industrial. Such a demand can be easily met using this modern biotechnology. However, the bioprocesses considered for these productions must be optimised to ensure maximum productivity or yield of the desired product. Such an approach is used to achieve the economic goal of producing large quantities of products at the lowest possible cost.

*Pichia pastoris* (syn. *Komagataella pastoris*) is a promising host for achieving high titres of recombinant proteins. It has been widely used in bioprocesses to produce several heterologous proteins. This host can overcome the limitations to produce recombinant proteins found in bacterial cells, such as *E. coli*. These limitations include insoluble expression, lack of post-translational modifications, and lack of disulphide bond formation [1]. Another factor to consider when choosing a suitable host is its ability to facilitate the secretion of the protein of interest, contributing to a high yield of the protein. All these limitations can be subjugated when using *P. pastoris* as the host and enable the secretion of correctly folded recombinant protein directly into the medium with very few endogenous proteins thereby, simplifying the downstream processing. *P. pastoris,* being a methylotrophic yeast, has the ability to utilize methanol as a sole carbon and energy source. The methanol utilisation plus phenotype (Mut^+^) is a widely used phenotype and facilitates high methanol concentrations in large scale fermentations. Since methanol induces the transcription level of alcohol oxidase (AOX genes), monitoring the concentration of this carbon source is often necessary to maximise protein production [2,3].

The consumption of natural sweeteners is estimated to reach a value of 28 million U.S. dollars by 2026 [4]. Thaumatin is a naturally derived sweet protein, which is currently gaining popularity in the market. This protein has a wide range of applications in the food industry, and therefore, can serve as a flavour enhancer, making it a desirable food ingredient [5]. It can be found in chewing gums, dairy products, pet foods and animal feed, as well as ice cream and sweets [6]. This is a globular protein found in *Thaumatococcus danielli* that is highly homologous [7] and obtained through aqueous extraction of the fruit. It includes six forms that are closely related (I, II, III, a, b, and c) and have similar molecular weights [7,8]. Thaumatin is a polypeptide of 207 amino acids and is highly soluble in water. The thermostability assessment of thaumatin in aqueous solutions has indicated that the protein remains stable in low pH ranges and can withstand heat treatments [9]. The stability of thaumatin is due to the tertiary structure of the protein consisting of eight disulphide bonds. 

Previous studies have shown the use of *P. pastoris* for the successful secretion of recombinant thaumatin [10,11,12,13]. Using the yeast α-factor prepropeptide sequence of *Saccharomyces cerevisiae*, the host organism can secrete the protein of interest directly into the media [11]. The recombinant protein can also be secreted from the host using its native signal sequence. For instance, Ide et al. [11] compared the effect of the native signal of the peptide on the final yield of the recombinant thaumatin secreted into the medium. The authors substantiate their findings by claiming that the production yield of thaumatin was influenced by the presence of N-terminal pre sequence. The secretion of recombinant thaumatin into the medium facilitates easier downstream processing and sample handling.

In bioprocessing, the interaction of various parameters determines the process development for achieving the product of interest. Investigating factors contributing to the efficient production of recombinant proteins is crucial to optimising the process. A key parameter to consider during recombinant protein production is the pH of the medium. It is altered or controlled depending on the protein of interest expected from the cultivation. Degradation of heterologous proteins produced during high cell density cultivation in the bioreactors can occur due to the increased concentration of proteases [14]. Proteolytic degradation of proteins, which is a major problem during heterologous protein production, occurs because of cell lysis. This is commonly noticed in high cell density fermentation, which can be due to the stresses caused by media components or more specifically, the pH of the media [15]. At a lower pH, proteolytic degradation can be reduced. For instance, Jahic et al. [16] have reported that serine protease activity was reduced when the cultivation was performed at a pH of 4.0 compared to 5.0 Therefore, adjusting the pH is vital to ensure the stability of the recombinant protein. It is worth noting that the optimal pH identified for the growth of the organism is not necessarily applicable for the desired recombinant protein. Charoenrat et al. [17] investigated the effect of pH on the production levels of endoglucanase and concluded that pH 5.0 is an optimal condition compared to 6.0 for large-scale production. The nature and ability of the recombinant protein function are determining factors in identifying the optimal pH value for production [18]. Since *P. pastoris* can grow within a wide range of pH, this facilitates the pH adjustment according to the requirement of the heterologous protein to be produced.

Another important environmental factor influencing recombinant protein production is temperature [19,20,21]. Past studies have indicated that for ensuring the correct protein folding and secretion, the environmental stress factors are to be considered [22]. Temperature, for instance, is one such parameter that can significantly influence protein folding. Dragosits et al. [23] summarise in their work that maintaining a lower temperature during cultivation facilitates effectual secretion of the heterologous protein. Ensuring a low temperature also reduces the chances of cell death and degradation of recombinant proteins [15,24]. Additionally, Dragosits et al. [23] reported that at a lower cultivation temperature, *P. pastoris* was able to showcase a three-fold increase in specific productivity.

Therefore, the objective of this study is to investigate the impact of cultivation, i.e., pH and temperature, on the growth of *P. pastoris* and the yield of recombinant thaumatin II. This work offers a first-time investigation in identifying the best culture conditions for maximising the thaumatin yield from *P. pastoris* GS115 and hence, aids as an initial screening step prior to further bioprocess optimisation. The knowledge acquired from such an approach can be easily scaled up for pilot-scale production, thereby, reducing the time required for process optimisation in large-scale recombinant thaumatin production.

## 2. Materials and Methods

### 2.1. Strain, Growth Media, and Chemicals

The pre-thaumatin-II-pro gene was cloned from *Thaumatococcus daniellii* and used to genetically modify *Pichia pastoris* GS115. VIB Protein Core (Zwijnaarde, Belgium) provided the wild-type and genetically engineered *P. pastoris* GS115 strains. The expression vector employing heterologous protein expression was pPICZalphaB, enabling the secretion of the protein directly into the media. The attained stable clone was then stored at −80 °C in glycerol stocks 5% (*w*/*v*) Yeast Peptone Dextrose (YPD, Carl Roth, Karlsruhe, Germany) broth and 25% (*w*/*v*) glycerol (99+ % p, Chem Lab, Zedelgem, Belgium) until the expression study.

### 2.2. Inoculum Preparation

The *P. pastoris* glycerol stock stored at −80 °C was used to prepare the preculture plate containing YPD medium (5% (*w*/*v*)), bacteriological agar (1.6% (*w*/*v*)) (VWR, Leuven, Belgium), and Zeocin (200 µg/mL) (InvivoGen, San Diego, CA, USA). After 72 h of incubation, three colonies from the preculture plate were transferred into 100 mL Erlenmeyer flasks containing 20 mL YPD broth (1% (w/v) yeast extract, 2% (*w*/*v*) peptone and 2% (*w*/*v*) dextrose) and 100 µg/mL of Zeocin.

### 2.3. Media Composition

In this study, six commonly used media formulations were used as described in the supplementary files (Tables S1 and S2) and the supplier information is indicated in Table S3. The media selected for the study were Basal salts medium (BSM), FM22, Modified Basal Salts medium (MBSM), d’Anjou, Buffered glycerol complex medium (BMGY) and Minimal glycerol medium (MGY) [25]. After autoclaving the respective media, the pH was adjusted to 5.0 and 6.0 by the addition of 20% ammonium hydroxide solution. From this, 40 mL was transferred to sterile baffled shake flasks and inoculated using 4% (*v*/*v*) of the prepared inoculum.

### 2.4. Optimisation of Cell Growth and Thaumatin Production in a Shake Flask

The *P. pastoris* GS115 strain with the pre-thaumatin-II-pro gene was cultivated in 40 mL of the respective media containing 1.0% (*w*/*v*) of glycerol at 30 °C and 220 rpm for 24 h to achieve biomass accumulation and later, transferred to a fresh medium of the same composition containing 1.0% (*v*/*v*) of methanol for 72 h for cell growth and production of the protein. The cultivations were performed in 100 mL baffled shake flasks for five days. Every day post-induction, 1 mL of methanol was supplemented to the media at specific time points, resulting in a concentration of 1.25% (*v*/*v*). The cell concentration was monitored throughout the experiment by taking four samples from the shake flask every two hours and plating the dilutions on Yeast extract peptone dextrose agar before methanol supplementation. Two samples were retrieved from the shake flasks each day prior to methanol supplementation to check the yield of thaumatin II. At the end of each cultivation, 1 mL of unfiltered sample from the shake flasks was transferred into pre-weighed 1.5 mL Eppendorf tubes to centrifuge and wash the cells for estimating the dry cell weight by drying at 55 °C for seven days.

### 2.5. Analytical Methods

Samples from the shake flasks were filtered using a 0.2 µm filter (Sarstedt, Nümbrecht, Germany) attached to a 10 mL syringe (BD Discardit™II, Fraga (Huesca), Spain) into an HPLC sample vial with a 150 µL insert (Fischer Scientific, Illkirch-Graffenstaden, France). The analysis was performed using an Agilent LC 1260 Infinity II system with a 1260 Infinity II diode-array detector HS (G7117C) (Agilent Technologies). The thaumatin separation was attained on Zorbax 300SB-C8 column, 4.6 × 150 mm, and 5 µm particle size (Agilent technologies, Santa Clara, CA, USA). The mobile phase consisted of Milli Q water (18.2 MΩ.cm at 25 °C, Millipore), ≥99.9% acetonitrile HPLC grade (Honeywell, Seelze, Germany), and 99+ % trifluoroacetic acid (Fischer Scientific, Merelbeke, Belgium). A linear gradient of 25% to 40% of acetonitrile containing 0.025% TFA in 30 min was used to attain a well-resolved peak. The detection of the protein was by absorbance at 196 nm. The protein peaks from the samples were compared using a thaumatin standard (Sigma Aldrich, Steinheim, Germany) and quantified from the calibration curve attained in the range of 9.37–300 mg/L.

Sodium dodecyl sulfate-polyacrylamide gel electrophoresis (SDS PAGE) analysis was conducted for the quantitative estimation of recombinant thaumatin II present in the media. To achieve this, stain-free gels containing 17.5% polyacrylamide and 1.0% (*w*/*v*) 2, 2, 2-trichloroethanol were hand cast. Prior to the analysis 50 µL of the filtered biological sample was mixed with 50 µL of sample buffer in a 1.5 mL Eppendorf tube. The mixture was heated to 95 °C for 5 min and placed in an ice-water bath. About 15 µL of the sample was loaded into each well and 5 µL of protein ladder (BioRad precision plus unstained) was loaded for comparison. The gel was run at 230 volts/cm for approximately one hour. The stain-free gels were activated on a Gel Doc Image system (BioRad) where the gels were exposed for 5 min. Using densitometric analysis the concentration of thaumatin was calculated based on the intensity observed and the thaumatin standard loaded within the range 9.37–150 mg/L.

### 2.6. Data Handling

The shake flask experiments were performed in pairs. Every sample was processed in duplicate, and each sample was injected into the HPLC twice. The raw data attained from the analysis were examined using the OpenLab CDS Chemstation Edition (version C.01.10, Agilent Technologies, Santa Clara, CA, USA). For the SDS PAGE analysis, each sample was tested twice in separate gels to ensure the reproducibility of the method used, and the mean value was used to calculate the protein concentration. All data processing was conducted in Microsoft Excel (Office 365), and the calculated values were expressed as mean and standard deviation.

## 3. Results and Discussion

The environmental conditions selected for fermentation can have a direct effect on cell growth, secretion yield of the recombinant protein and product stability within the medium. Hence, it is vital to assess these conditions in a screening step prior to further optimisation and upscaling of the fermentation process. In this research, a comparison was performed to study the influence of the pH of the growth medium in the methanol induction phase on the secretion yield of recombinant thaumatin II. Furthermore, the temperature effect on the growth of the host organism and its influence on the protein yield was also evaluated. A recombinant *Pichia pastoris* GS115 strain capable of secreting recombinant thaumatin II into the growth medium was used in this study. Firstly, the influence of pH (5.0 and 6.0) was studied using commonly used *P. pastoris* media formulations, such as BMGY, FM22, BSM, MBSM, MGY and d’Anjou. Following the pH study, two media were selected to assess three temperature levels (20, 25 and 30 °C) to investigate the optimal value with respect to recombinant thaumatin II secretion into the medium.

### 3.1. Effect of Induction pH on Growth of Pichia pastoris

**Figure 1:**
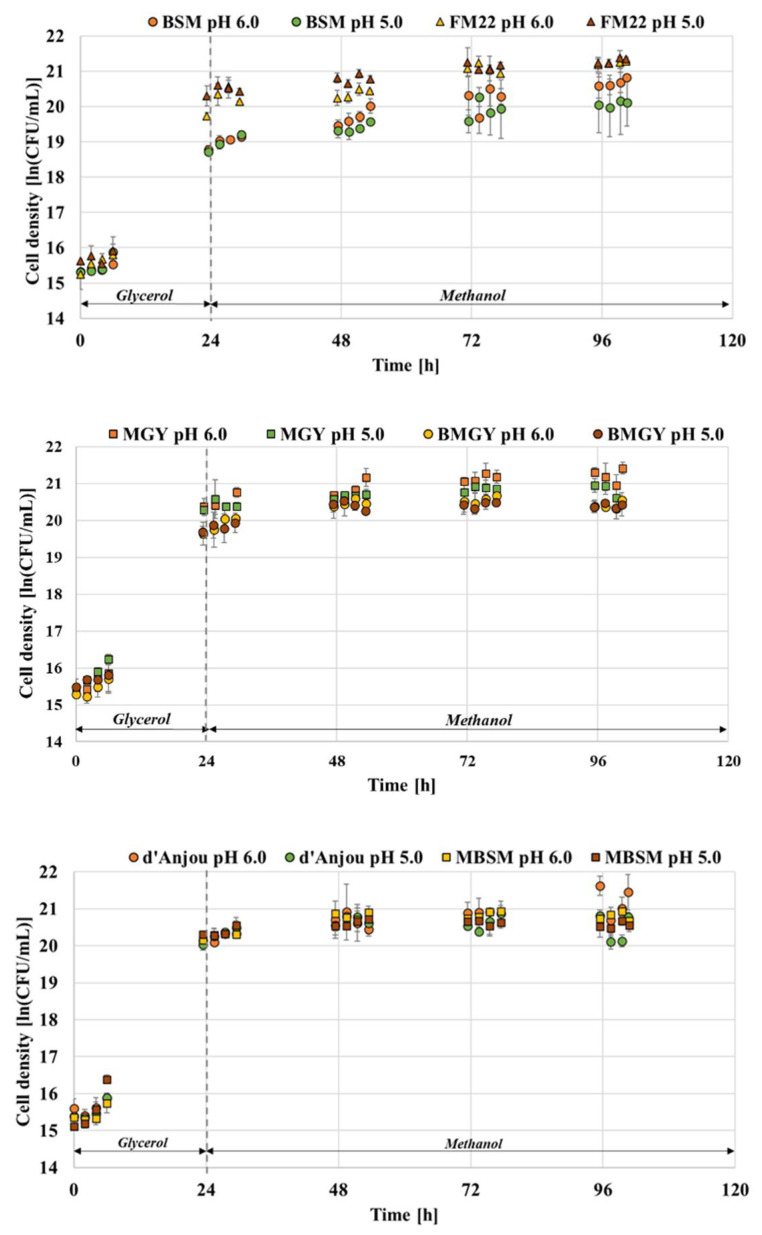
Growth comparison of *P. pastoris* at pH 5.0 and 6.0 when grown in commonly used fermentation media at 30 °C.

Monitoring the viability and growth pattern of the cells is vital in understanding how the adopted cultivation strategies affect yeast physiology and secretion yield. The *P. pastoris* GS115 Mut + strain capable of secreting thaumatin was grown in six different media formulations namely, BMGY, FM22, BSM, MBSM, MGY and d’Anjou at pH 5.0 and 6.0 to compare the growth of the host organism. Figure 1 indicates the growth of *P. pastoris* when grown in the respective media formulations at pH values of 5.0 and 6.0.

The media formulations recommended for *P. pastoris* cultivation include chemically defined and complex types. Especially for large-scale high cell density cultivations, the most recommended formulations are of chemical nature. Such a medium can guarantee a better batch-to-batch consistency and is often a cheaper alternative compared to complex media [26]. However, from the growth results, it was observed that BSM, one of the most recommended formulations for *P. pastoris* fermentation was seen to exhibit a lower viable cell density at pH 6.0 after 96 h of fermentation compared to the rest of the media. The final viable cell density achieved for BSM was approximately 20.07 ln (CFU/mL), which is lower than the FM22 medium which resulted in 21.30 ln (CFU/mL). In comparison to all the media assessed in this study, the FM22 medium resulted in the highest viable cell density at end of the fermentation [25]. Since *P. pastoris* can grow at a range of pH values, it is worthwhile to assess its influence on the growth of the host organism and the production yield of the protein. Most of the fermentation associated with this host system suggests maintaining the induction pH at either 5.0 or 6.0. From the current study, the media comparison at two different pH values has led to some interesting findings. A comparison between MGY and BMGY media was performed, and it was observed that the former showed a higher viable cell density at the end of fermentation with 21.15 ln CFU/mL. Upon lowering the pH to 5.0, the cell density for MGY decreased to 20.83 ln CFU/mL; in the case of the BMGY medium, the cell densities remained the same at both pH 6.0 and 5.0, accounting for 20.36 and 20.40 ln CFU/mL, respectively.

When comparing the cell growth between the two pH values, it was observed for BSM medium that the viable cell density attained at pH 5.0 was lower than at pH 6.0 resulting in 20.07 ln (CFU/mL) and 20.67 ln (CFU/mL), respectively. The FM22 medium, on the other hand, showed similar final cell densities of 21.30 and 21.24 ln (CFU/mL) at both pH values. Both d’Anjou and MBSM media exhibited lower cell densities than FM22 at the end of fermentation. When the pH effect was assessed for these media, a lower cell density was observed for both compositions at a lower pH. For MBSM, the viable cell density of 20.79 ln (CFU/mL) at pH 6.0 dropped to 20.55 ln (CFU/mL) at pH 5.0. Similarly, the cell density of 21.19 ln (CFU/mL) attained for the d’Anjou medium at pH 6.0 decreased to 20.45 ln (CFU/mL) at pH 5.0.

**Figure 2:**
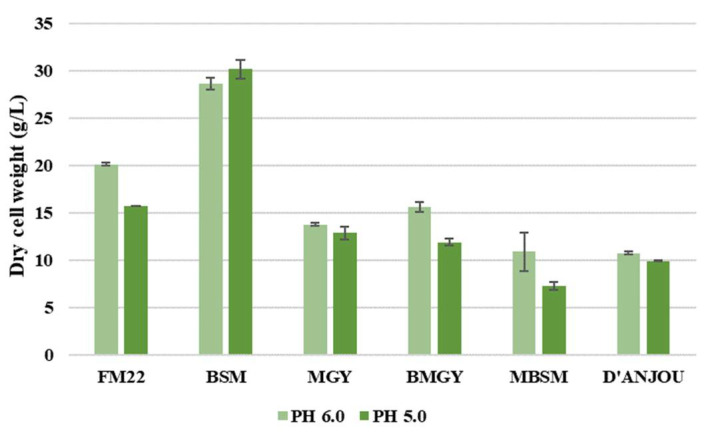
Dry cell weight comparison of *P. pastoris* at pH 6.0 and 5.0 when grown in different media formulations at 30 °C.

Furthermore, a comparison of the dry cell weight (DCW) was performed to assess the influence of pH change on the total cell density. As illustrated in Figure 2, almost all media compositions showed a higher DCW when the yeast was grown at pH 6.0, except for BSM. BSM resulted in the lowest viable cell density among all media formulations, as can be seen from comparing the viable cell densities for the different media in Figure 1. On the other hand, BSM exhibited the highest DCW at both pH values. Since the DCW includes both viable and dead cells, the high DCW in combination with a low viable cell count for BSM implies considerable cell death during fermentation. The study from Zhao et al. [27] elaborates that the BSM causes high osmotic pressure thereby reducing the growth and viability of *P. pastoris*. The authors also address in the study that the long duration of the fermentation can lead to lower cell viability for some strains causing cell death, an increase of proteases and degradation of the recombinant protein secreted into the media. After BSM, the medium that is exhibiting a higher viable cell density and DCW is FM22, which resulted in 1.3 times higher DCW at pH 6.0 when compared to pH 5.0. Similarly, BMGY and MBSM displayed a higher DCW value when grown at pH 6.0. The DCW results attained for the MBSM were in line with the viable cell density. However, the effect of pH on DCW in BMGY was the opposite of the effect on viable cell density in the same medium, where the latter remained unchanged. Although the DCW values resulting from pH 6.0 were higher for most media, MGY and d’Anjou media did not display drastic changes in the cell density. In conclusion, the pH comparison has demonstrated that a pH of 6.0 exhibits ideal growth of *P. pastoris* than 5.0 and is, therefore, identified as the optimal pH for the fermentation.

### 3.2. Effect of Induction pH on Recombinant Thaumatin II Production

To optimise the bioproduction conditions, it is interesting to know the effect of pH on the production levels of the recombinant thaumatin II. *P. pastoris* can grow within a wide range of pH values from 3.0 to 7.0 [28]. However, the optimal pH value to produce the protein may vary depending on the type of strain, genetic modification performed, and type of recombinant protein preferred. Since the solubility of different proteins depends on the selected pH, it is vital to evaluate the effect of pH on recombinant protein production [29]. Thaumatin, for instance, is a basic protein that has stability between 2.7 and 6.0 [5]. In this study, pH 5.0 and 6.0 were evaluated during the methanol induction phase to determine whether the protein production is enhanced when the pH is lowered during the induction phase compared to the growth phase. Furthermore, for more precise comparison of both pH values on the production levels, a combined effect of pH and media formulation is investigated.

When the secretion of recombinant proteins is considered, the proteins are distributed between three main locations, i.e., cytoplasm, periplasm, or cultivation medium [30]. Often, secretion is preferred for recombinant protein production because it facilitates a simpler purification with improved protein folding compared to periplasmic and cytoplasmic productions. However, secretion into the media can also pose some issues making the purification more complex. Importantly, the stability of the protein secreted into the medium can be compromised if appropriate cultivation conditions are not selected. Since most of the *P. pastoris* fermentations are performed within the range of pH 5.0 to 6.0, it is worthwhile to assess if these values affect the protein secretion into the media.

While assessing the levels of protein production from the six media at a pH of 5.0 and 6.0, as shown in Table 1, the highest production of recombinant thaumatin II, i.e., approximately 62.79 mg/L was observed for the BMGY medium when grown at pH 6.0. The second-best medium based on the production yield of thaumatin II was exhibited by FM22 at pH 6.0 resulting in a concentration of 43.29 mg/L followed by BSM at pH 6.0 resulting in 42.77 mg/L. It has been established in some studies that lowering pH aids in the inhibition of neutral proteases [31] leading to a possible increase in protein levels in the media. Past studies have pointed out the positive impact on production levels of recombinant proteins when the induction pH is lowered [32,33]. However, the results attained from this study indicated otherwise. For all the media evaluated in this study, the thaumatin II secretion levels were higher when the induction pH was maintained at 6.0. According to the data indicated in Table 1, the highest difference in the protein levels was noticed for the BMGY medium, where the secretion levels decreased by about 15 mg/L when a lower pH was used. Additionally, in the d’Anjou medium, a high relative decrease of about 32% occurs at a pH of 5.0 compared to 6.0. The differences observed in the final production levels of thaumatin in the remaining media were comparatively low between the two pH values. In the case of MBSM, the final levels attained were well below the detectable levels at the induction of pH 5.0. Interestingly, the difference in secretion levels at 48 h and 72 h is much less between the two pH levels compared to the final secretion level. This indicates that the effect of pH on protein production is more related to the maximum protein production yield that can be obtained and less to the rate of production.

**Figure 3:**
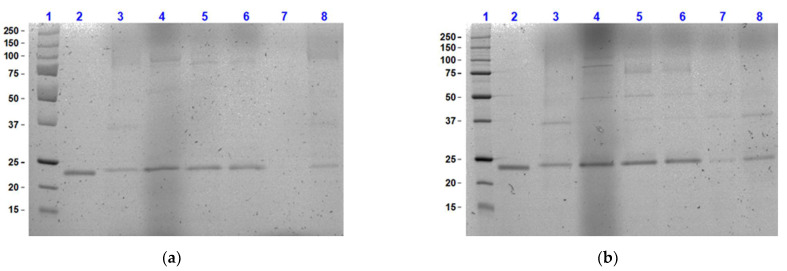
SDS PAGE analysis performed for detection of thaumatin at pH (**a**) 5.0 and (**b**) 6.0 when grown at 30 °C. Lane 1: Bio-Rad precision plus unstained (kDa); Lane 2: Thaumatin standard 37.5 mg/L; Lane 3: MGY medium; Lane 4: BMGY medium; Lane 5: BSM; Lane 6: FM22 medium; Lane 7: MBSM; Lane 8: d’Anjou medium.

By performing an SDS PAGE analysis the thaumatin results at pH 5.0 and 6.0 were confirmed. It was seen from the sample analysis as shown in Figure 3, that the pH adjustment did not substantially affect the secretion of protein at the end of fermentation for most media. However, quantified values from densitometric analysis as shown in Table 2 confirmed that maintaining the culture medium at pH 6.0 exhibited at least a slightly higher yield for all the media formulations. These results also confirm the strongest decrease in thaumatin production when changing the pH of the BMGY medium from 6.0 to 5.0. From the study conducted by Calık et al. [34], it was identified that the cell concentration in the culture improved at a pH of 6.0. However, while their study was conducted with a *P. pastoris* Mut^+^ strain as well, the production levels of the recombinant protein, human growth hormone, were the highest at a pH value of 5.0. This pattern was not observed in the current study. Both the cell density and recombinant protein production yield observed at pH 5.0 were lower than in the experiments conducted at pH 6.0. Based on the effects of pH on cell growth and thaumatin secretion, BMGY and FM22 media were shown to be the most promising media, and therefore, were selected to assess the influence of temperature in the following section.

### 3.3. Effect of Induction Temperature on Growth of Pichia pastoris

**Figure 4:**
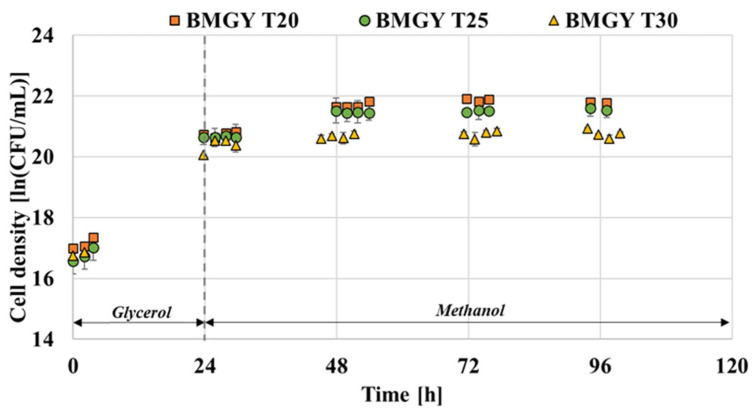

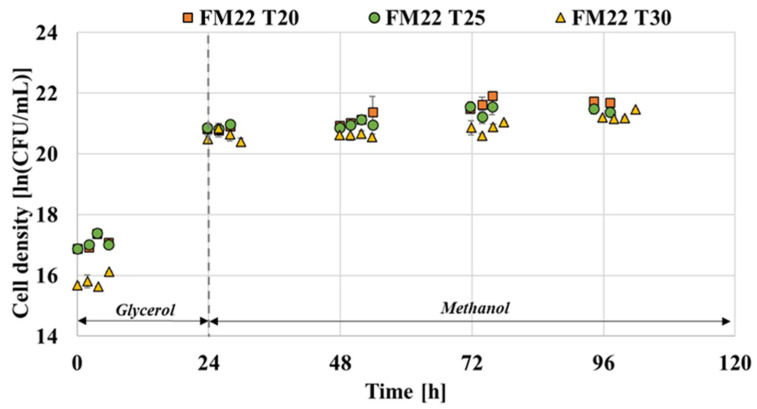
Effect of temperature on the growth of *P. pastoris* grown on FM22 and BMGY media with an induction pH of 6.0.

It is highlighted in numerous studies that lowering the temperature enables the effective reduction of cell death [15,16,24]. Therefore, the effect of temperature on the growth of *P. pastoris* GS115 capable of producing thaumatin was assessed. The advantages of low cultivation temperature include the favourable effects on energy metabolism, protein folding and secretion; protein degradation and aggregation in recombinant microorganism cells [24,35,36,37]. As illustrated in Figure 4, the growth pattern of *P. pastoris* when grown in FM22 and BMGY media is compared at the three levels of temperature. Both media exhibited a lower cell density at the end of fermentation when grown at 30 °C compared to 20 and 25 °C. This is in accordance with the findings of Li et al. [24] where the viable cell densities were seen to drop after three days of fermentation at 30 °C compared to 23 °C. The viable cell densities attained for FM22 medium at 20, 25 and 30 °C were 21.72, 21.43 and 21.15 ln (CFU/ mL), respectively. In the case of the BMGY medium, the cell densities attained were 21.79, 21.56 and 20.76 ln (CFU/mL) for 20, 25 and 30 °C, respectively. In the case of both media, as observed in Figure 4, *P. pastoris* grown at 30 °C reached the stationary phase faster than the cells at 20 and 25 °C. This observation is in line with the findings of Li et al. [24] where *P. pastoris* X-33 strain grown at 30 °C exhibited faster growth compared to 23 °C.

**Figure 5:**
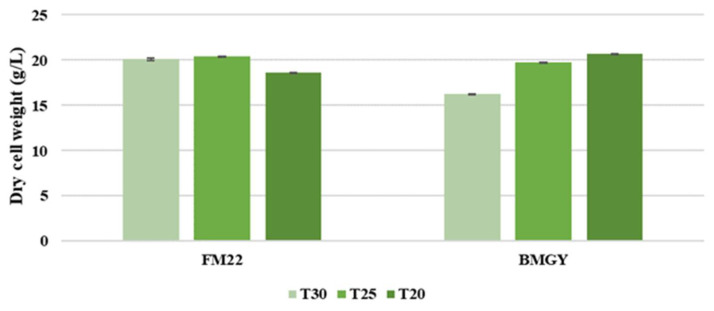
Dry cell weight comparison of *P. pastoris* at temperatures 20, 25 and 30 °C when grown in FM22 and BMGY media with an induction pH of 6.0.

Zhong et al. [38] found that elevated temperature would prolong the accumulation process of the nascent proteins in the endoplasmic reticulum (ER), leading to the overload of the ER and cell death eventually. Hence, lowering the temperature is beneficial for cell viability and the folding capacity of the ER [38]. A DCW comparison was conducted to assess the influence of temperature on both media formulations. As observed in Figure 5, the DCW was higher for the FM22 medium when grown at 25 and 30 °C and decreased when cultivated at a lower temperature of 20 °C. On the contrary, for BMGY medium DCW value was higher at low temperatures of 20 and 25 °C. In the study conducted by Jiang et al. [39], the lowering of temperature from 30 to 25 °C contributed to higher cell densities in both shake flasks and bioreactors when a chemically defined medium was used.

### 3.4. Effect of Induction Temperature on Recombinant Thaumatin II Production

One of the major bottlenecks limiting the secretion yield is the retention of the protein within the host cells due to limited folding. Inadequate folding can lead to the translocation of the protein back to the cytosol and result in degradation [40]. To accommodate an easier secretion, the upregulation of unfolded protein response (UPR) is key to identifying endoplasmic reticulum (ER) stress, thereby reducing the burden of misfolded protein in the ER. One of the factors regulating the UPR is the cultivation conditions and hence, optimising them helps in alleviating the degradation of the protein. Certain proteins, such as psychrophilic and mesophilic proteins require low temperatures to ensure high production. Furthermore, lower temperature poses the benefit of reducing proteolytic degradation. There have been studies on improving the secretion yield of the protein by lowering the cultivation temperature. It is worth investigating this phenomenon prior to optimising the production of thaumatin as factors, such as cell physiology and specific productivity can be regulated by environmental conditions [41].

Two well-performing media, FM22 and BMGY, showcasing high thaumatin II secretion yield were selected for assessing the effect of temperature. Studying a chemically defined and complex medium will help in investigating whether the medium plays a role in the production yield of the protein in combination with the selected temperatures. The commonly used temperature for *P. pastoris* fermentation is 30 °C which is also maintained during the induction for protein expression. From the experimental results, it was observed that lowering the temperature failed to enhance the secretion of the protein into the medium (Table 3). For both media, the thaumatin secretion levels were reduced at lower temperatures. Especially at 20 °C, the thaumatin levels observed were too low in comparison to the yield attained at 30 °C. This is in accordance with the study of Jiang et al. [39], where lowering the temperature did not exhibit a higher protein yield of recombinant manganese peroxidase (MnP). On the other hand, in the study of Jariyachawalid et al. [42], lowering the fermentation temperature from 30 to 25 °C resulted in higher D-phenylglycine aminotransferase (D-PhgAT) activity yields from *P. pastoris* strain KM_AT3_ELS10. However, in this study, it was seen that the production levels decreased again when the temperature was lowered from 25 to 20 °C. Hong et al. [20] highlight in their study that low cultivation temperature facilitates proper protein folding and investigated temperatures ranging from 10 to 30 °C. However, a decrease in total soluble protein was observed when the cultivation temperature dropped to 20 °C.

The growth temperature influences the cellular processes of *P. pastoris*. For example, the temperature will affect the central carbon metabolism, stress response and protein folding [23]. Lowering the temperature has shown improvements in the yield of some proteins. Hence, it is relevant to identify the factors contributing to some of the bottlenecks involved in the protein expression. Some studies have indicated the advantage of lowering the fermentation temperature to diminish cell death and protein degradation. For example, when the production levels of the 3H6 Fab fragment were assessed, lowering the growth temperature from 30 °C to 20 °C resulted in a three-fold increase in the specific productivity [23]. Hence, the lowering of temperature demonstrated a significant improvement in the yield of the protein secreted into the medium. Temperature can have an influence on the regulation of the unfolded protein response (UPR). For instance, from the study of Zhong et al. [38], it was observed that lowering the cultivation temperature from 30 to 20 °C aided in the upregulation of the UPR. The upregulation can increase the yield of the protein due to faster processing of the recombinant product within the ER [38,43]. A lower temperature is expected to reduce proteolysis and unfolding of the recombinant protein. The study of Li et al. [24] also highlights the influence of temperature on the production yield of recombinant herring antifreeze protein (hAFP). It was seen from this work that the yield of the protein improved significantly when the medium temperature was lowered from 30 to 23 °C. The authors pointed out that protein degradation was visible when the expression temperature was 30 °C but lowering the temperature to 23 °C eliminated the problem. Furthermore, Jahic et al. [16] deduced from their experimental study that a temperature-limited fed-batch (TLFB) process resulted in higher AOX activity compared to a methanol-limited fed-batch (MLFB) process. The results corroborated by their study are directed toward the advantage of lowering the culture temperature which dramatically decreases the protease activity.

While previous research led to the expectation of an increased protein yield at decreased temperatures, this study showed that the highest levels of thaumatin II were secreted at 30 °C. At the end of the fermentation, there was a clear decrease in thaumatin II yield for every 5 °C temperature decrease. Table 3 provides an overview of the data from the study conducted using BMGY and FM22. An interesting aspect that emerged from the analysis is that the thaumatin present in the media degraded over time when produced at 20 °C. For instance, comparing the levels of protein in BMGY and FM22 at 20 °C, the resulting yield after 100 h of fermentation was 8.53 mg/L and 2.32 mg/L, respectively, a value slightly lower than what was achieved after 72 h. However, this trend was absent when the growth temperature was 25 or 30 °C. Although the protein levels increased over time during the fermentation at 25 °C, the most optimal temperature to attain a high thaumatin II secretion was observed at 30 °C. In the case of fermentation using FM22 medium, the increasing trend of thaumatin II levels at 25 °C was lower than in BMGY medium. At 30 °C, the fermentations of 72 h already outperform the 100 h fermentations at lower temperatures. During the last 28 h, there is a further increase in the thaumatin level by about 18 mg/L for both media. Hence, the protein production was confirmed to be optimal at 30 °C.

**Figure 6:**
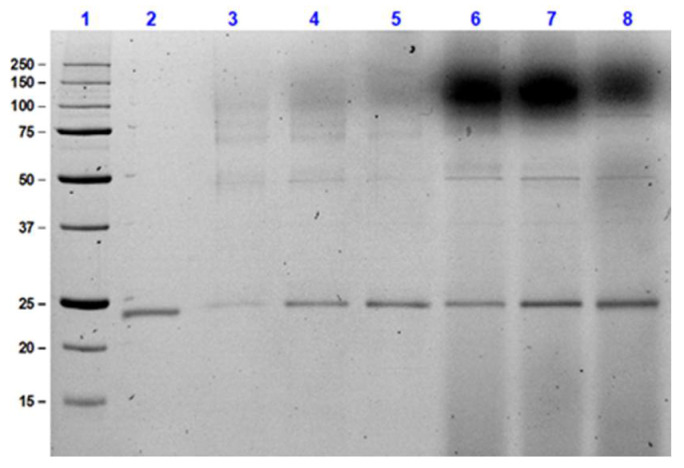
SDS PAGE analysis of recombinant thaumatin expression using 17.5% SDS-PAGE gel. Lane 1: Bio-Rad precision plus unstained (kDa); Lane 2: Thaumatin standard 37.5 mg/L; Lane 3: FM22 medium at 20 °C; Lane 4: FM22 medium at 25 °C; Lane 5: FM22 medium at 30 °C; Lane 6: BMGY medium at 20 °C; Lane 7: BMGY medium at 25 °C and Lane 8: BMGY medium at 30 °C. All samples measured are from the 100th hour of fermentation.

An SDS PAGE was performed to confirm the data attained from the chromatographic analysis. The results observed from the SDS PAGE as indicated in Figure 6 aligned with the values attained from HPLC. At lower cultivation temperatures of 20 and 25 °C, the secretion yield of recombinant thaumatin II at the end of *P. pastoris* fermentation was detected as faint bands on the gel. Using densitometric analysis these bands were quantified as indicated in Table 4. In comparison to the results attained from both HPLC and PAGE, the highest yield of recombinant thaumatin was, therefore, confirmed at 30 °C, and similar values were determined for the two media using both techniques.

## 4. Conclusions

Recombinant protein production in *Pichia pastoris* can be limited by the complex secretory pathway due to the challenges it imposes on the process optimisation. Furthermore, the secretion yield of the protein can be greatly compromised by the intracellular processes which are directly influenced by the culture conditions selected during the cultivation. Hence, it is vital to know the culturing conditions that optimize bioproduction. This study is aimed at investigating the influence of pH and temperature on the growth of *P. pastoris* and the secretion yield of recombinant thaumatin II. In the current study, the production of recombinant thaumatin II was found to be influenced by external factors and operating conditions, such as media components, temperature, and pH. The culture conditions selected for this study, i.e., pH, and temperature were seen to influence both the growth of the *P. pastoris* Mut^+^ strain and the secretion levels of recombinant pre-pro thaumatin II in the media. To assess the influence of pH, fermentations were performed for six different media formulations commonly used in *P. pastoris* cultivation and were evaluated at pH 5.0 and 6.0 while maintaining the temperature at 30 °C. From the acquired data, it was observed that secretion levels for thaumatin were higher when grown at a pH of 6.0 and that the highest yields were achieved for BMGY, FM22 and BSM at 62.79, 43.29 and 42.77 mg/L of thaumatin II, respectively. The temperature of the culture medium during the induction of protein expression is identified as a crucial factor that influences the productivity of the cells in heterologous protein production. To assess the influence of temperature on the *P. pastoris* growth and secretion yield of thaumatin II, induction phases were performed at 20, 25 and 30 °C with an induction pH of 6.0 for the most promising media, i.e., BMGY and FM22. These experiments demonstrated the highest levels of thaumatin at 30 °C and a clear decrease with decreasing temperatures. Based on these results, it can be concluded that the optimal culturing conditions are achieved at a pH of 6.0 and a temperature of 30 °C. Interestingly, the results demonstrated that the trends observed from the qualitative effects of both temperature and pH occurred in all the media that were investigated.

## Figures and Tables

**Table 1:** Concentration levels of recombinant thaumatin II secreted into the media at pH 5.0 and 6.0 when grown in commonly used fermentation media at 30 °C and quantified using HPLC.

pH	Fermentation Time [h]	Thaumatin Concentration [mg/L]					
		BSM	FM22	MGY	BMGY	MBSM	d’Anjou
5.0	48	8.85	9.99	N.D.	12.09	N.D.	N.D.
	72	22.37	23.86	11.10	30.10	N.D.	11.96
	100	39.50	40.00	12.21	47.14	N.D.	12.55
6.0	48	9.24	8.84	N.D.	14.13	N.D.	12.72
	72	25.43	25.08	11.00	37.62	N.D.	14.80
	100	42.77	43.29	15.17	62.79	11.17	18.47

N.D.: Not detectable.

**Table 2:** Concentration of thaumatin quantified at the end of fermentation using SDS PAGE densitometric analysis.

pH	Thaumatin Concentration [mg/L]					
	BSM	FM22	MGY	BMGY	MBSM	d’Anjou
5.0	38.57	39.02	13.52	49.70	N. D.	14.15
6.0	39.53	41.29	16.43	67.43	10.24	17.30

**Table 3:** Concentration levels of recombinant thaumatin II secreted into the media with an induction pH of 6.0 and grown at 20, 25 and 30 °C as quantified using HPLC.

Media	Temperature [°C]	Fermentation Time [h]	Thaumatin Concentration [mg/L]
BMGY	20	72	9.54
		100	8.53
	25	72	21.66
		100	38.34
	30	72	44.62
		100	62.79
FM22	20	72	2.88
		100	2.32
	25	72	10.62
		100	11.02
	30	72	25.08
		100	43.29

**Table 4:** Concentration levels of recombinant thaumatin II secreted into the media of induction pH 6.0 and grown at 20, 25 and 30 °C as quantified using SDS PAGE densitometric analysis at the end of fermentation.

Media	Temperature [°C]	Thaumatin Concentration [mg/L]
BMGY	20	13.48
	25	36.35
	30	60.38
FM22	20	6.14
	25	15.09
	30	42.84

## Data Availability

All data is available without restrictions upon request through contact with the corresponding author.

## Supplementary Materials

The following supporting information can be downloaded at: https://www.mdpi.com/article/10.3390/foods11101438/s1, Table S1: Media formulations used for *Pichia pastoris* fermentation involving BSM, FM22, MGY, BMGY, d’Anjou and MBSM medium; Table S2: Supplementary salt formulations PTM1, PTM4, trace solution 1 and trace solution 2 used for BSM, FM22, MBSM and d’Anjou medium, respectively; Table S3: List of chemicals and their suppliers used in the media formulation for *Pichia pastoris* fermentation (.docx).

## References

[1] Rudolph R., Lilie H. (1996). In vitro folding of inclusion body proteins. FASEB J..

[2] Macauley-Patrick S., Fazenda M.L., McNeil B., Harvey L.M. (2005). Heterologous protein production using the *Pichia pastoris* Expression System. Yeast.

[3] Cregg J.M. (2007). Introduction: Distinctions between *Pichia pastoris* and other expression systems. Methods Mol. Biol..

[4] Wunsch N.-G. Sugar Substitutes and Sweeteners in the U.S.—Statistics & Facts, 2021. Statista. www.statista.com.

[5] Joseph J.A., Akkermans S., Nimmegeers P., Van Impe J.F. (2019). Bioproduction of the recombinant sweet protein thaumatin: Current state of the art and Perspectives. Front. Microbiol..

[6] Smith J., Hong-Shum L. (2011). Food Additives Data Book.

[7] Van der Wel H., Loeve K. (1972). Isolation and characterization of Thaumatin I and II, the sweet-tasting proteins from *Thaumatococcus daniellii* Benth. Eur. J. Biochem..

[8] Ledeboer A.M., Verrips C.T., Dekker B.M.M. (1984). Cloning of the natural gene for the sweet-tasting plant protein thaumatin. Gene.

[9] Younes M., Aquilina G., Castle L., Engel K.H., Fowler P., Frutos Fernandez M.J., Fürst P., Gürtler R., EFSA Panel on Food Additives and Flavourings (FAF) (2021). Re-evaluation of thaumatin (E 957) as food additive. EFSA J..

[10] Masuda T., Tamaki S., Kaneko R., Wada R., Fujita Y., Mehta A., Kitabatake N. (2004). Cloning, expression and characterization of recombinant sweet-protein thaumatin II using the methylotrophic Yeast *Pichia pastoris*. Biotechnol. Bioeng..

[11] Ide N., Masuda T., Kitabatake N. (2007). Effects of pre- and pro-sequence of thaumatin on the secretion by *Pichia pastoris*. Biochem. Biophys. Res. Commun..

[12] Masuda T., Ide N., Ohta K., Kitabatake N. (2010). High-yield secretion of the recombinant sweet-tasting protein thaumatin I. Food Sci. Technol. Res..

[13] Healey R.D., Lebhar H., Hornung S., Thordarson P., Marquis C.P. (2017). An improved process for the production of highly purified recombinant thaumatin tagged-variants. Food Chem..

[14] Zhang Y., Liu R., Wu X. (2007). The proteolytic systems and heterologous proteins degradation in the methylotrophic yeast *Pichia pastoris*. Ann. Microbiol..

[15] Sinha J., Plantz B.A., Inan M., Meagher M.M. (2004). Causes of proteolytic degradation of secreted recombinant proteins produced in methylotrophic yeast *Pichia pastoris*: Case study with recombinant ovine interferon-τ. Biotechnol. Bioeng..

[16] Jahic M., Wallberg F., Bollok M., Garcia P., Enfors S.O. (2003). Temperature limited fed-batch technique for control of proteolysis in *Pichia pastoris* bioreactor cultures. Microb. Cell Factories.

[17] Charoenrat T., Khumruaengsri N., Promdonkoy P., Rattanaphan N., Eurwilaichitr L., Tanapongpipat S., Roongsawang N. (2013). Improvement of recombinant endoglucanase produced in *Pichia pastoris* KM71 through the use of synthetic medium for inoculum and ph control of proteolysis. J. Biosci. Bioeng..

[18] Inan M., Chiruvolu V., Eskridge K.M., Vlasuk G.P., Dickerson K., Brown S., Meagher M.M. (1999). Optimization of temperature–glycerol–ph conditions for a fed-batch fermentation process for recombinant hookworm (*Ancylostoma caninum*) anticoagulant peptide (ACAP-5) production by *Pichia pastoris*. Enzym. Microb. Technol..

[19] Laurent P., Buchon L., Guespin-Michel J.F., Orange N. (2000). Production of pectate lyases and cellulases by *Chryseomonas luteola* strain MFCL0 depends on the growth temperature and the nature of the culture medium: Evidence for two critical temperatures. Appl. Environ. Microbiol..

[20] Hong F., Meinander N.Q., Jönsson L.J. (2002). Fermentation strategies for improved heterologous expression of laccase in *Pichia pastoris*. Biotechnol. Bioeng..

[21] Woo J.H., Liu Y.Y., Stavrou S., Neville D.M. (2004). Increasing secretion of a bivalent anti-t-cell immunotoxin by *Pichia pastoris*. Appl. Environ. Microbiol..

[22] Mattanovich D., Gasser B., Hohenblum H., Sauer M. (2004). Stress in recombinant protein producing yeasts. J. Biotechnol..

[23] Dragosits M., Stadlmann J., Albiol J., Baumann K., Maurer M., Gasser B., Sauer M., Altmann F., Ferrer P., Mattanovich D. (2009). The effect of temperature on the proteome of recombinant *Pichia pastoris*. J. Proteome Res..

[24] Li Z., Xiong F., Lin Q., d’Anjou M., Daugulis A.J., Yang D.S.C., Hew C.L. (2001). Low-temperature increases the yield of biologically active herring antifreeze protein in *Pichia pastoris*. Protein Exp. Purif..

[25] Joseph J.A., Akkermans S., Van Impe J.F. (2022). Effect of media composition on the growth of *Pichia pastoris* and secretion levels of recombinant thaumatin II. BioTeC+, Chemical and Biochemical Process Technology and Control, Department of Chemical Engineering, KU Leuven, 9000 Ghent, Belgium.

[26] Matthews C.B., Kuo A., Love K.R., Love J.C. (2017). Development of a general defined medium for *Pichia pastoris*. Biotechnol. Bioeng..

[27] Zhao H.L., Xue C., Wang Y., Yao X.Q., Liu Z.M. (2008). Increasing the cell viability and heterologous protein expression of *Pichia pastoris* mutant deficient in PMR1 gene by culture condition optimization. Appl. Microbiol. Biotechnol..

[28] Ahmad M., Hirz M., Pichler H., Schwab H. (2014). Protein expression in *Pichia pastoris*: Recent achievements and perspectives for heterologous protein production. Appl. Microbiol. Biotechnol..

[29] Borhan N.M., Maleki A., Ahmadi H., Sharifat S.A., Nejati M., Norouzian D. (2011). Evaluation of pH/buffering conditions effect on the optimization of Recombinant Human Erythropoietin expression in the methylotrophic yeast, *Pichia pastoris*. J. Pharm. Health Sci..

[30] Ma Y., Lee C.-J., Park J.-S. (2020). Strategies for optimizing the production of proteins and peptides with multiple disulfide bonds. Antibiotics.

[31] Wang X., Shen X., Zhao H., Sun Y., Liu T., Liu Y., Xu L., Yan Y. (2011). Combined strategies for the improvement of heterologous expression of a His-tagged Yarrowia lipolytica lipase Lip2 in *Pichia pastoris*. Afr. J. Biotechnol..

[32] Koubaa M., Barba F.J., Roohinejad S. (2021). Fermentation Processes.

[33] Jahic M., Gustavsson M., Jansen A.-K., Martinelle M., Enfors S.-O. (2003). Analysis and control of proteolysis of a fusion protein in *Pichia pastoris* fed-batch processes. J. Biotechnol..

[34] Çalık P., Bayraktar E., İnankur B., Soyaslan E.Ş., Şahin M., Taşpınar H., Açık E., Yılmaz R., Özdamar T.H. (2010). Influence of pH on recombinant human growth hormone production by *Pichia pastoris*. J. Chem. Technol. Biotechnol..

[35] Georgiou G., Valax P. (1996). Expression of correctly folded proteins in *Escherichia coli*. Curr. Opin. Biotechnol..

[36] Cassland P., Jönsson L.J. (1999). Characterization of a gene encoding trametes versicolor laccase A and improved heterologous expression in *Saccharomyces cerevisiae* by decreased cultivation temperature. Appl. Microbiol. Biotechnol..

[37] Zepeda A.B., Pessoa A., Farías J.G. (2018). Carbon metabolism influenced for promoters and temperature used in the heterologous protein production using *Pichia pastoris* yeast. Braz. J. Microbiol..

[38] Zhong Y., Yang L., Guo Y., Fang F., Wang D., Li R., Jiang M., Kang W., Ma J., Sun J. (2014). High-temperature cultivation of recombinant *Pichia pastoris* increases endoplasmic reticulum stress and decreases production of human interleukin-10. Microb. Cell Factories.

[39] Jiang F., Kongsaeree P., Schilke K., Lajoie C., Kelly C. (2008). Effects of ph and temperature on recombinant manganese peroxidase production and stability. Biotechnol. Fuels Chem..

[40] Xie W., Ng D.T.W. (2010). Erad substrate recognition in budding yeast. Semin. Cell Dev. Biol..

[41] Gasser B., Maurer M., Rautio J., Sauer M., Bhattacharyya A., Saloheimo M., Penttilä M., Mattanovich D. (2007). Monitoring of transcriptional regulation in *Pichia pastoris* under protein production conditions. BMC Genom..

[42] Jariyachawalid K., Laowanapiban P., Meevootisom V., Wiyakrutta S. (2012). Effective enhancement of pseudomonas stutzeri D-phenylglycine aminotransferase functional expression in *Pichia pastoris* by co-expressing *Escherichia coli* GroEL-GroES. Microb. Cell Factories.

[43] Raschmanová H., Weninger A., Knejzlík Z., Melzoch K., Kovar K. (2021). Engineering of the unfolded protein response pathway in *Pichia pastoris*: Enhancing production of secreted recombinant proteins. Appl. Microbiol. Biotechnol..

