# Supplementary material for: Effects of Temperature and pH on Recombinant Thaumatin II Production by Pichia pastoris
Source: Foods. 2022 May 16;11(10):1438. doi: 10.3390/foods11101438 (PMC9141780; doi:10.3390/foods11101438)
Supplement: Supplementary file 1 [file foods-11-01438-s001.zip › MDPI_Foods_Supplementary materials.pdf]

Effects of Temperature and pH on Recombinant Thaumatin II Production by *Pichia pastoris*

Jewel Ann Joseph , Simen Akkermans and Jan F. M. Van Impe *

BioTeC+, Chemical and Biochemical Process Technology and Control, Department of Chemical Engineering, KU Leuven, 9000 Ghent, Belgium
*Correspondence: Jan F. M. Van Impe, **jan.vanimpe@kuleuven.be**;

COMPONENTS	BSM (1 L)	FM22 (1 L)	MBSM (1 L)	d'Anjou (1 L)	BMGY (1 L)	MGY (1 L)
Phosphoric acid, 85 %	26.7 mL	-	-	-	-	-
Calcium sulphate	0.93 g	1.0 g	-	-	-	-
Potassium sulphate	18.2 g	14.3 g	-	-	-	-
Magnesium sulphate heptahydrate	14.9 g	11.7 g	3.2 g	4.7 g	-	-
Potassium hydroxide	4.13 g	-	-	-	-	-
Glycerol	1.0 g	1.0 g	1.0 g	1.0 g	1.0 g	1.0 g
Monopotassium phosphate	-	42.9 g	10.0 g	12.0 g	-	-
Calcium chloride dihydrate	-	-	0.35 g	0.36 g	-	-
Ammonium sulphate	-	5.0 g	-	20.0 g	-	-
Yeast Extract	-	-	-	-	10.0 g	-
Bacto peptone	-	-	-	-	20.0 g	-
Phosphate buffer pH 6.0	-	-	-	-	1.0 M	1.0M
Yeast nitrogen base	-	-	-	-	13.4 g	13.4 g
Biotin	5 x10^{-5} g	5 x10^{-5} g	5 x10^{-5} g	5 x10^{-5} g	5 x10^{-5} g	5 x10^{-5} g
PTM1 salts	4.0 mL	-	-	-	-	-
PTM4 salts	-	4.0 mL	-	-	-	-
Trace solution 1	-	-	4.3 mL	-	-	-
Trace solution 2	-	-	-	1.0 mL	-	-

Table S1: Composition of the different media used for *Pichia pastoris* fermentation

COMPONENTS	PTM1 (1 L)	PTM4 (1 L)	TRACE SOLUTION 1 (1 L)	TRACE SOLUTION 2 (1 L)
Cupric sulfate pentahydrate	6.0 g	2.0 g	6.0 g	-
Sodium iodide	0.08 g	0.08 g	-	-
Manganese sulfate	3.0 g	3.0 g	3.0 g	0.159 g
Sodium molybdate dihydrate	0.2 g	0.20 g	1.0 g	0.071 g
Boric acid	0.02 g	0.02 g	0.01 g	0.026 g
Cobalt chloride	0.5 g	0.5 g	-	-
Zinc chloride	20.0 g	7.0 g	-	-
Ferrous sulfate heptahydrate	65.0 g	22.0 g	65.0 g	-
Sulfuric acid	5.0 mL	1.0 mL	98.0 mL	-
Calcium sulfate dihydrate	-	0.50 g	-	0.007 g
Zinc sulfate heptahydrate	-	7.0 g	20.0 g	0.621 g
Potassium iodide	-	-	0.42 g	0.044 g
Ferric chloride hexahydrate	-	-	-	1.579 g

Table S2: Composition of trace elements supplemented in different media

Table S3:List of chemicals and their suppliers used in the media formulation for *Pichia pastoris* fermentation.

Component	Manufacturer
YPD	Carl Roth
Bacteriological agar	VWR chemicals
Bacto peptone	Becton Dickinson
Bacto yeast extract	Becton Dickinson
YNB	Carl Roth
D-glucose	Acros Organics
Zeocin	Bio-Connect B.V. Netherlands
Glycerol	Chem Lab Analytical
Methanol	VWR Chemicals
Monopotassium phosphate	Fisher Chemical
Biotin	Sigma Aldrich
Dipotassium hydrogen phosphate	VWR Chemicals
Sulfuric acid	Fisher Chemicals
Ammonium hydroxide	Carl Roth
Sodium chloride	Sigma Aldrich
Chloroform	Sigma Aldrich
Phosphoric acid (85%)	Chem-Lab Analytical
Calcium sulphate	Sigma Aldrich
Potassium sulphate	Chem-Lab Analytical
Magnesium sulphate heptahydrate	Chem-Lab Analytical
Potassium hydroxide	Fisher Chemical
Ammonium sulphate	Chem-Lab Analytical
Calcium chloride dihydrate	VWR Chemicals
Ferric chloride hexahydrate	Acros Organics
Zinc sulphate heptahydrate	VWR Chemicals
Manganese sulphate monohydrate	Merck
Potassium iodide	Carl Roth
Boric acid	Acros Organics
Sodium molybdate dihydrate	Carl Roth
Cupric sulphate pentahydrate	Merck
Sodium iodide	Fisher Chemicals
Cobalt chloride	Riedel-de Haen
Zinc chloride	Acros Organics
Ferrous sulphate heptahydrate	HoneyWell
